# Overview of the Molecular Mechanism of Bacterial Environmental Adaptation by Comprehensive Analysis

**DOI:** 10.3390/ijms24087602

**Published:** 2023-04-20

**Authors:** Tomohiro Shimada, Hideji Yoshida

**Affiliations:** 1 School of Agriculture, Meiji University, Kawasaki 214-8571, Kanagawa, Japan; 2 Department of Physics, Osaka Medical and Pharmaceutical University, Takatsuki 569-8686, Osaka, Japan

So far, the genome sequences of more than tens of thousands of organisms have been determined, and the overall picture of the genes that make up one organism has been clarified [https://www.ncbi.nlm.nih.gov/genome (accessed on 1 April 2023)]. However, even in *Escherichia coli*, the model organism with the most accumulated knowledge, there are still many genes with unknown physiological functions on the genome, and in addition, there are many unclear mechanisms for adapting to environmental changes [1,2,3]. Understanding the functional information of all genes and the biological mechanism based on the comprehensive genome regulation mechanism is an important task in life science in the 21st century. Bacteria are suitable model organisms today because they have a wealth of functional information on individual genes, can be analyzed for their entire genome using current comprehensive analysis methods, and can provide a complete picture of the life system.

In addition, although many genome sequences have been uncovered in higher organisms, such as animals and plants, revealing the specific functions of interesting genes and proteins, their roles and physiological significance for the whole organism are still difficult to understand. In contrast, microorganisms for which many gene functions have been elucidated provide a complete picture of the organism. This allows for a more accurate understanding of the physiological roles and molecular mechanisms of the subject under study [4]. For example, if one is trying to understand the pathogenicity of a microorganism, studying only specific virulence factors will not lead to a deeper understanding. This is because virulence factors are not always expressed in microorganisms but are expressed in adaptation to a certain environment or a certain cellular condition [5,6,7]. Microorganisms are also often used to produce useful compounds, but metabolic pathways do not always maintain the same activity; they change their activities in response to changing environments or cellular conditions [8,9,10]. In short, organisms are often studied for specific functions for specific purposes but understanding them not only from the analysis of specific factors related to those purposes but also in terms of their roles for the organism, their cooperative relationships with cells, and their regulatory network mechanisms, will provide essential understanding and applications.

The new Special Issue entitled “Overview of the Molecular Mechanism of Bacterial Environmental Adaptation by Comprehensive Analysis” of the International Journal of Molecular Sciences includes a total of seven contributions: six original articles and one review, providing new information about the overview of the molecular mechanism of bacterial environmental adaptation.

Under certain stress conditions, the presence or absence of certain key genes determines the host’s adaptation to that environment. The bile resistance of intestinal bacteria is among the key factors responsible for their successful colonization of and survival in the mammalian gastrointestinal tract. Bile resistance is primarily mediated by bile salt hydrolase (BSH) enzymes. Understanding the function of this enzyme and analyzing its conservation in the gut microbiota is important for understanding the effects of probiotics on human health, defined as “live microbial feed supplements which beneficially affect the host animal by improving its intestinal microbial balance”. Morinaga et al. demonstrated that lactate-producing Atopobiaceae bacteria isolated from mouse intestines showed high resistance to mammalian bile extracts due to significant bile salt hydrolase (BSH) activity [11]. They also isolated BSH proteins from *Leptogranulimonas caecicola* and *Granulimonas faecalis* and performed functional analysis, concluding that these BSH enzymes confer bile resistance. This study expands the current understanding of the phylogenetic diversity of BHS-producing probiotic candidates in mammalian digestive tracts. Furthermore, their findings suggest that BSH-producing *Atopobiaceae* bacteria and their BSH enzymes with broad substrate specificity could be regarded as new targets for future probiotic research.

*Acinetobacter* is a genus of aerobic chemoorganotrophic saprophyte bacteria. Strains of *Acinetobacter* are among the most ubiquitous bacteria and live in a wide variety of ecological niches. Some strains, predominantly *A. baumannii*, are causative agents of nosocomial infections such as septicemia, pneumonia, meningitis, urinary tract infections, skin infections, gastroenteritis and wound infections, as well as infectious diseases in animals. One of the most important questions facing researchers is the question about the mechanisms underlying the amazing plasticity of representatives of the genus *Acinetobacter* and their ability to adapt to a wide range of living conditions. The review by Maslova et al. summarizes the data on the mechanisms of development of the adaptability of *Acinetobacters* to various living conditions in the environment and in the clinic [12]. In particular, the plasmids of modern strains are enriched with antibiotic-resistant genes, while the content of genes involved in resistance to heavy metals and arsenic is comparable to plasmids from modern and ancient strains. They claimed that many of the plasmids of *Acinetobacter* could be mobilized and that the widespread megaplasmids can not only move on their own but also mobilize other plasmids, the necessary genes are readily available in all *Acinetobacter* strains. Then, there is a selection of options to be adapted depending on the given living conditions. They concluded that the adaptability of *Acinetobacter* is provided not only by their high metabolic potential encoded in the chromosome but to a large extent by their plasmids, although this contribution can obviously be different in different species.

In bacterial communities, species that can rapidly escape predation by phagotrophic protists, for example, via phenotypic plasticity, have a competitive advantage under high predation pressure. Protist grazing effectively controls bacterial biomass and enables the transport of a substantial part of the bacterial biomass to higher trophic levels through the “microbial loop”. Therefore, bacteria–protist trophic interactions have profound consequences for organic matter and energy flow, as well as food-web structure in aquatic systems. Furthermore, in natural systems, it has been shown that predator-driven escape mechanisms are a key driver in shaping bacterial communities in aquatic environments. The escape behavior might thereby be triggered by the recognition of protist predators via chemical cues. Villalba et al. investigated the escape response of the marine bacterium *Marinobacter adhaerens* in the presence of either planktonic (nanoflagellate) or surface-associated (amoeba) protist predators, following population dynamics [10]. They demonstrated the presence of contrasting defensive mechanisms in the chemotaxis mutant *M. adhaerens* towards planktonic or surface-attached predators in the two main pelagic habitats: water and particle surfaces. They found multiple plastic responses (i.e., escape into the predator-free habitat and cell clumping) against predator grazing; furthermore, bacterial defense responses differed in response to the planktonic or surface-attached predator. The presence of active predator escape mechanisms affects species interactions and community composition with potential consequences for the flux of energy and matter through the microbial loop. Their study highlights that taking microbial interactions into account might be crucial to assess how changing abiotic conditions via nutrient availability and particle concentration will impact energy transport and remineralization in aquatic systems.

In their natural environment, many bacterial species form a biofilm after attaching to a solid surface. Biofilms allow bacteria to grow and survive in conditions of stress. The Gram-negative bacterium *Escherichia coli* and the related species *Salmonella* spp. produce amyloid fibers, known as “curli fimbriae”, which are the major protein component of the extracellular matrix. Curli fimbriae are amyloids that are involved in solid surface adhesion and bacterial aggregation during biofilm formation. In the model microorganism *Escherichia coli (E. coli)*, the curli protein CsgA is coded by a *csgBAC* operon gene, and the transcription factor CsgD is essential to induce its curli protein expression. Aside from controlling curli protein expression, CsgD regulates the expression of at least 20 other genes; these include genes of unknown function that were predicted to be associated with curli fimbriae formation. Among these proteins, YccT is induced by CsgD, which directly binds to the 35–58 base pairs located upstream of the yccT transcription start site. However, the role of YccT remains largely unknown. Sano et al. revealed that YccT inhibits the formation of curli fimbriae and, on the other hand, functions as a regulator of OmpR phosphorylation [7]. In their conclusion, their data demonstrate that YccT is a novel inhibitor of curli formation by inhibiting both CsgA polymerization and curli gene expression. Thus, they propose to rename YccT to CsgI (curli synthesis inhibitor) as an inhibitor of curli synthesis.

Flagella motility helps bacteria reach favorable environments and is vital in substrate adhesion, biofilm formation, and virulence processes. Bacterial flagellum synthesis genes form an ordered cascade in which the expression of one gene at a given level requires the transcription of another gene at a higher level. This regulatory cascade includes three gene classes. In *Escherichia coli*, class I genes form the *flhDC* master operon at the top of the hierarchy, encoding an FlhDC transcriptional activator of class II gene expression. Most class II genes encode flagella export system and basal body components. The *fliA* gene at this second level encodes a sigma factor, σ^28^ (or RpoF), specific for flagella genes. σ^28^ and the anti-sigma factor FlgM positively and negatively regulate class III operons, respectively. However, the genome-wide transcriptional regulation of FlhDC was poorly understood. Takada et al. attempted to re-examine the role of FlhDC, the master regulator of flagellum formation genes, in the entire *E. coli* genome regulatory network by using Genomic SELEX (gSELEX)-chip screening to uncover a direct set of target genes in vitro [8]. They identified novel target genes involved in the sugar utilization phosphotransferase system, sugar catabolic pathway of glycolysis, and other carbon source metabolic pathways in addition to the known flagella formation target genes. Based on further experimental results, they proposed that the flagella master transcriptional regulator FlhDC acts in the activation of a set of flagella-forming genes, sugar utilization, and carbon source catabolic pathways to provide coordinated regulation between flagella formation, operation and energy production. Recently, it was estimated that all the flagella on a cell consume approximately 10% of the intercellular ATP produced by *E. coli*. The results of this study revealed a rational microbial mechanism that cooperatively activates the sugar catabolic pathway during flagellar formation and motility in order to produce the energy associated with it.

The growth of bacteria in a natural environment is often limited, so within these environments, they subsequently enter a stationary phase. The long-term survival of bacteria under stressful conditions depends on the establishment of various stress defense systems. For example, the stationary phase adaptation of bacteria is accompanied by marked changes in their gene expression patterns. The genes required for the response to various stresses and for survival in the stationary phase have been identified by comprehensive analyses using gene deletion or overexpression strains. At present, however, even within the model bacterium *E. coli*, the function of one-fourth of the species is still unknown because most stress response genes are not expressed within laboratory conditions. Accordingly, the full set of regulators involved in the expression of stress conditions during the stationary phase has not yet been identified. In the absence of knowledge of transcription factors (TFs) and the conditions affecting the expression of regulatory functions, the ordinary in vivo approach is not useful in identifying regulatory targets of hitherto uncharacterized TFs, as TFs are not always expressed. To understand the whole picture of the mechanisms of environmental adaptation of microorganisms, a comprehensive understanding of the role of transcriptional regulators and the genome-wide and transcriptional regulators involved in the regulation of genes that are key genes involved with environmental adaptation is necessary. To elucidate the function of the functionally unknown transcription factor YgfI in *E. coli*, Kobayashi et al. performed gSELEX-chip screening to identify all YgfI regulatory targets on the *E. coli* genome [6]. Based on the experimental results, it was proposed to rename YgfI as SrsR (a stress response regulator in the stationary phase) since YgfI target genes were involved in biofilm formation, hydrogen peroxide resistance, and antibiotic resistance, and most of them were expressed in the stationary phase. Their study reveals one aspect of the newly stationary-phase adaptation mechanism through the transcriptional regulation of microorganisms.

When an organism is exposed to stress, the expression of many genes is regulated at transcriptional and translational levels. For instance, in Gram-negative bacteria, such as *Escherichia coli*, the sigma factor RpoD binds to RNA polymerase under favorable growth conditions and serves as a basic transcription mechanism. However, when growth is arrested in response to stress, such as nutrient starvation, the anti-sigma factor Rsd is expressed and binds to the global regulator RpoD, which becomes inactivated. At the translational level, ribosome modulation factor (RMF), which is expressed in response to growth arrest, binds to 70S ribosomes to form inactive 100S ribosomes (dimers of 70S ribosomes), which regulate translational activity. Thus, bacteria exposed to stress survive by regulating the expression of several genes at the transcriptional and translational levels. In addition, stress due to fluctuations in the concentration of metal ions essential for various intracellular pathways is regulated by a homeostatic mechanism involving metal-responsive transcription factors. Yoshida et al. examined the binding of a few metal-responsive TFs to the promoter regions of *rsd* (encoded anti-major sigma factor) and *rmf* (encoded ribosome modulation factor) through promoter-specific TF screening and studied the effects of these TFs on the expression of *rsd* and *rmf* in each TF [9]. The results suggest that several metal-responsive TFs (CueR, Fur, KdpE, MntR, NhaR, PhoP, ZntR, and ZraR) and metal ions (Cu^2+^, Fe^2+^, K^+^, Mn^2+^, Na^+^, Mg^2+^, and Zn^2+^) influence *rsd* and *rmf* gene expression while regulating transcriptional and translational activities. Their results are important for understanding bacterial survival strategies under stress conditions and provide new insights into countermeasures for diseases in which stress tolerance is an issue.

Thus, this Special Issue consisted of papers describing the importance of many factors for microbial adaptation to the environment, their importance among microbial species and their relationship to various cellular functions and behaviors, as well as their regulatory networks. In this post-genome era, it is necessary to continue to comprehensively analyze and understand interesting factors, whether it is their role in the cell as a whole or their function or regulatory network, using microorganisms that have accumulated genetic and functional information as a single organism.

## References

[1] Tantoso E., Eisenhaber B., Sinha S., Jensen L.J., Eisenhaber F. (2023). About the dark corners in the gene function space of *Escherichia coli* remaining without illumination by scientific literature. Biol. Direct..

[2] Gagarinova A., Hosseinnia A., Rahmatbakhsh M., Istace Z., Phanse S., Moutaoufik M.T., Zilocchi M., Zhang Q., Aoki H., Jessulat M. (2022). Auxotrophic and prototrophic conditional genetic networks reveal the rewiring of transcription factors in *Escherichia coli*. Nat. Commun..

[3] Ishihama A., Shimada T. (2021). Hierarchy of transcription factor network in *Escherichia coli* K-12: H-NS-mediated silencing and Anti-silencing by global regulators. FEMS Microbiol. Rev..

[4] Ishihama A. (2018). Building a complete image of genome regulation in the model organism *Escherichia coli*. J. Gen. Appl. Microbiol..

[5] Femerling G., Gama-Castro S., Lara P., Ledezma-Tejeida D., Tierrafría V.H., Muñiz-Rascado L., Bonavides-Martínez C., Collado-Vides J. (2022). Sensory Systems and Transcriptional Regulation in *Escherichia coli*. Front. Bioeng. Biotechnol..

[6] Kobayashi I., Mochizuki K., Teramoto J., Imamura S., Takaya K., Ishihama A., Shimada T. (2022). Transcription Factor SrsR (YgfI) Is a Novel Regulator for the Stress-Response Genes in Stationary Phase in *Escherichia coli* K-12. Int. J. Mol. Sci..

[7] Sano K., Kobayashi H., Chuta H., Matsuyoshi N., Kato Y., Ogasawara H. (2023). CsgI (YccT) Is a Novel Inhibitor of Curli Fimbriae Formation in *Escherichia coli* Preventing CsgA Polymerization and Curli Gene Expression. Int. J. Mol. Sci..

[8] Takada H., Kijima K., Ishiguro A., Ishihama A., Shimada T. (2023). Genomic SELEX Reveals Pervasive Role of the Flagella Master Regulator FlhDC in Carbon Metabolism. Int. J. Mol. Sci..

[9] Yoshida H., Shimada T., Ishihama A. (2023). Metal-Responsive Transcription Factors Co-Regulate Anti-Sigma Factor (Rsd) and Ribosome Dimerization Factor Expression. Int. J. Mol. Sci..

[10] Villalba L.A., Kasada M., Zoccarato L., Wollrab S., Grossart H.P. (2022). Differing Escape Responses of the Marine Bacterium *Marinobacter adhaerens* in the Presence of Planktonic vs. Surface-Associated Protist Grazers. Int. J. Mol. Sci..

[11] Morinaga K., Kusada H., Tamaki H. (2022). Bile Salt Hydrolases with Extended Substrate Specificity Confer a High Level of Resistance to Bile Toxicity on Atopobiaceae Bacteria. Int. J. Mol. Sci..

[12] Maslova O., Mindlin S., Beletsky A., Mardanov A., Petrova M. (2022). Plasmids as Key Players in *Acinetobacter* Adaptation. Int. J. Mol. Sci..

